# Safer Sleep Guidance in Standalone UK Smartphone Apps for Parents of Newborns and Infants: Systematic mHealth App Review

**DOI:** 10.2196/95642

**Published:** 2026-07-10

**Authors:** Senna Yousef, Niamh Meaney, Dawn Dowding, Norina Gasteiger

**Affiliations:** 1 Division of Nursing, Midwifery and Social Work University of Manchester Manchester, England United Kingdom; 2 The School of Health and Society University of Salford Salford, England United Kingdom

**Keywords:** babies, digital health, guidance, infants, mHealth, mobile health, newborn, review, safer sleep

## Abstract

**Background:**

Parents are increasingly using smartphone apps to help guide decision-making around aspects of caregiving, including safer sleep. However, it remains unclear whether the guidance provided aligns with best practice guidelines.

**Objective:**

This review aimed to identify smartphone apps that provide safer sleep guidance and to assess their content, technical features, functionality, and quality. A secondary aim was to make recommendations on the improvements that existing and future apps can make to provide safer sleep guidance.

**Methods:**

In January 2026, we searched the UK Google Play Store and Apple App Store for safer sleep guidance apps for parents and caregivers of newborns and infants (babies aged ≤12 months). Information regarding content, technical features, and functional features was extracted and summarized descriptively. Each app was evaluated by 2 raters using the Mobile App Rating Scale (MARS) and the IMS Institute for Healthcare Informatics functionality score. Adherence to best practice sleep guidance was assessed using bespoke criteria. Interrater reliability was calculated.

**Results:**

A total of 1345 apps were identified, with 12 meeting the inclusion criteria. All were free to download, with 42% (5/12) containing in-app purchases. Only 17% (2/12) were affiliated with a professional health, medical, clinical, or government body. All 12 apps contained some information regarding safer sleep practices, although this was addressed and named differently (eg, calling it cot death, sudden infant death syndrome, or safe sleep). Of the 9 criteria relating to safer sleep best practice guidance, the apps mentioned an average of 6.75 (SD 1.71; range 3-9) criteria. Some promoted unsafe sleep practices (ie, around swaddling and cosleeping). The apps included an average of 5.4 of the 11 IMS functionality criteria (SD 2.5; range 2-9). The mean MARS score was 3.7 out of 5 (SD 0.43; range 2.92-4.13), and most (11/12, 92%) had a minimum acceptability score of 3 or above. Two had been formally trialed or tested. Although the language used was considered accessible (easy to understand), none of the apps allowed the language to be changed (beyond English), and very few (3/12, 25%) enabled users to change to night/dark mode, which would be considered essential when using these apps at night.

**Conclusions:**

Several apps about safer baby sleep exist. Few discuss safer sleep guidance comprehensively or raise awareness about sudden infant death syndrome and its links with unsafe sleep. All the reviewed apps met the minimum acceptability criteria for quality; however, not many were formally trialed or tested. More long-term research should be conducted on the effectiveness of safer sleep apps on adherence to safer sleep guidance. We recommend that apps relating to infant and baby safety in general include information on realistic safer sleep practices that are informed by best practice guidelines and take a risk minimization approach.

## Introduction

Safer sleep refers to a range of evidence-based practices that reduce the risk of sudden infant death syndrome (SIDS), an ongoing and significant public health concern. SIDS is defined as the sudden, unexpected, and unexplained death of an otherwise healthy infant occurring in babies aged 12 months or younger and most commonly within the first 6 months [1]. SIDS is listed in the *ICD-11* (*International Classification of Diseases, 11th Revision*) under the code MH11: sudden infant death syndrome and includes terms such as “cot death,” “infantile sudden infant death syndrome,” and related variants [2]. As the exact etiology of SIDS remains unknown and is believed to result from a combination of factors, current guidance focuses on implementing measures, such as safer sleep practices, that mitigate and reduce associated risks [1]. In the United Kingdom, safer sleep recommendations are primarily informed by guidance from The Lullaby Trust, the National Health Service (NHS), and the National Institute for Health and Care Excellence (NICE), all of which aim to reduce the incidence of SIDS.

The safest sleep environment for an infant is a separate sleep space, located within the same room as their carer [3]. Infants should be placed on their backs on a firm, flat, waterproof mattress, with their feet positioned closest to the foot of the cot or Moses basket. The cot or Moses basket should be kept free of objects and contain only the mattress and lightweight bedding [3-5]. They should be kept smoke-free, and the room temperature should be maintained between 16 °C and 20 °C, as overheating is a contributing factor to SIDS [3,6]. Current guidance recommends room sharing with their carer for at least the first 6 months [4]. This is central advice provided by professionals, and all parents should be given risk-reduction information on bed-sharing to support informed decision-making [6].

Parents should be advised not to bed-share if their baby has a low birth weight, weighs less than 2.5 kg, was born prematurely, or if either parent has consumed 2 or more units of alcohol, smokes, is taking medication that causes drowsiness, or has used recreational drugs [3,6]. Cosleeping should never occur on a sofa or armchair, as this significantly increases the risk of fatal sleep accidents and SIDS [7]. Parents should also assess the sleep environment for potential entrapment risks, including any gaps between the mattress and the bedframe or adjacent walls [8].

According to provisional data from the Office for National Statistics [9], a total of 164 unexplained infant deaths occurred in England and Wales in 2023, representing 7.1% of all infant mortality. Of these, 52% were classified as SIDS, equating to 0.14 sudden infant deaths per 1000 live births in 2023. Evidence indicates that hazardous sleep environments are present in most unexplained infant deaths in the United Kingdom, which is a key focus of national guidance [10].

While safer sleep campaigns, notably the “Back to Sleep” initiative launched in the 1990s, contributed to a 90% reduction in SIDS in England, this decline has plateaued in recent years [10,11]. Although the campaign was successful in targeting position-related SIDS, it has had less impact on deaths associated with cosleeping, which now accounts for approximately 30%-50% of all SIDS cases, mostly due to hazardous cosleeping environments, such as parental alcohol or drug consumption (which may cause drowsiness), smoking, preterm infants, or sofa or armchair use [3,10]. Additionally, didactic approaches that rely on fear, judgment, or inflexible ideal rules are often ineffective. Many parents report struggling to follow advice they perceive as unrealistic, particularly during periods of routine disruption [11]. In contrast, risk-reduction approaches acknowledge real-world circumstances and provide practical strategies that explain how and why safer sleep practices protect infants [11].

Messaging of safer sleep campaigns must be adapted and reinforced for the current generation of parents, as gaps remain in parental knowledge and adherence to safer sleep guidance, particularly in higher-risk groups. A cross-sectional survey conducted by Pease et al [12] in deprived areas of Bristol, United Kingdom, found that mothers in higher-risk groups were less likely to breastfeed, had more difficulty recalling 2 or more unprompted correct SIDS risk reduction strategies, and scored lower overall on prompted safer sleep scenarios. Notably, only 52% of the 400 mothers surveyed identified infant sleep position as a risk reduction strategy for SIDS [12].

In May 2024, UK adult smartphone users averaged 38 apps on their devices, and 32.5 million visited one of the top 10 health and well-being websites or apps [13]. Of the adult visitors to NHS websites that month, 57% were women [13]. Mobile health (mHealth) apps, which leverage the widespread use of smartphones, provide a cost-effective way to deliver accessible information and influence specific, health-related behaviors within targeted populations [14]. Additionally, with the increase in artificial intelligence (AI), many people, including parents, are turning to AI for information surrounding a multitude of topics; however, there are concerns about AI models such as ChatGPT because they draw their information from what is available on the internet, leading to the potential for misleading or incorrect data [15].

Technology, specifically mHealth apps, is increasingly influencing how parents make decisions about infant sleep. Lambert et al [11] found that most of their participants (28 families) relied on online resources, including sleep tracking apps, social media, and search engines, to guide their understanding of infant sleep, particularly when they perceived contact with health visitors was limited. Younger parents reported a stronger reliance on online sources and influencer content, preferring social media platforms over the NHS website, which they described as overly wordy or jargon-heavy. mHealth apps offer an opportunity to bridge the gap left by traditional services by providing resources that are collaboratively developed, accessible, and succinct, which can help parents plan for safer sleep and navigate routine disruptions, rather than relying solely on traditional didactic approaches [11].

Research surrounding systematic reviews of publicly and commercially available mHealth apps specifically targeting safer sleep for newborns and infants has not yet been conducted in the United Kingdom, although some research does exist on similar topics. For example, Simon et al [16] conducted a content analysis of smartphone apps for children’s sleep; however, they reviewed apps that offer sleep improvement strategies rather than safer sleep guidance for babies and infants. Virani et al [17] reviewed mHealth apps targeting new parents, specifically parents of newborns and infants; however, the study focused on apps that provided sleep tracking, sleeping aids, and photo sharing, and their target sleeping aid applications presented white noise and lullabies to soothe infants to sleep. They did not provide education on safer sleep guidance. Another review by Lee-Tobin et al [18] examined the educational information provided in sleep mHealth apps; however, it was not solely targeted toward newborns and infants. A final review by Grigsby-Toussaint et al [19] excluded apps targeting babies and included those used as sleep alarms. This suggests a gap in the availability of systematic evaluations of mHealth apps targeting parents of infants and newborns that provide guidance on safer sleep practices.

Safer sleep guidance is widely available on the internet, although it is difficult to access evidence-based, reliable guidance via an app without knowing the names of specific organizations or apps that provide this information. This is further emphasized by the increase in misinformation surrounding safer sleep on various social media platforms [20], making it difficult for parents of newborns, especially first-time parents, to access trustworthy information. This systematic mHealth app review will therefore seek to answer the question of what stand-alone safer sleep apps are available for parents and caregivers of newborns and infants in the United Kingdom and what their quality and functionality are. How accessible are they, and do they adhere to best practice guidelines?

## Methods

### Review Design

A systematic review of safer sleep mHealth apps available on the UK Google Play (Android) and Apple App Store (iOS) was undertaken. This review was conducted using the 7-step process, and the review question was informed by the TECH (target user, evaluation focus, connectedness, and health domain) framework [21] (Textbox 1). The protocol is registered on the Open Science Framework [22], and the review was reported using an initial version of the CAPPRRI (Consensus for APP Review Reporting Items) guidance [23], which was under development at the time of the review. Multimedia Appendix 1 contains the completed CAPPRRI checklist.

**The TECH (target user, evaluation focus, connectedness, and health domain) framework used to determine the review question.**

**Target user**

- Parents and caregivers of newborns up to infancy

**Evaluation focus**

- Quality, functionality, accessibility, and adherence to best practice guidelines

**Connectedness**

- Stand-alone

**Health domain**

- Safer sleep

This review was conducted by a UK pediatric nursing student in her second year of study (SY) and a UK midwifery student in her second year of study (NM), under the guidance of 2 researchers with extensive experience in conducting systematic mHealth app reviews and digital health evaluation (NG and DD). One author is also a registered nurse (DD). The clinical training backgrounds of the reviewers (SY and NM) may have positively influenced the appraisal of app content, particularly with respect to infant safety, clinical credibility, and alignment with evidence-based guidance. To mitigate any potential biases (eg, favoring mHealth interventions), all stages of the review followed a predefined protocol, used explicit inclusion criteria, and applied standardized and reproducible appraisal methods.

### Eligibility Criteria, Search, and Selection

As seen in Table 1, the eligibility criteria were determined through application of the TECH framework [21]. The target users were parents or caregivers of newborns and infants (0-12 months), including individuals and families, while apps targeting health care professionals, nurseries, care providers, or organizations were excluded. For the purpose of this review, infants were defined as children aged 12 months or younger, including the neonatal period (0-28 days) and postneonatal period (aged ≤12 months) [9]. The evaluation focus included app quality, functionality, accessibility, and adherence to best practice guidelines; privacy features were excluded because personal data were not expected to be collected. Eligible apps were required to be stand-alone and specifically address safer sleep by providing educational or informational content. Apps that functioned solely as sleep trackers, feeding or milestone trackers, or sound machines were excluded. Apps that cost to download were also excluded.

**Table 1:** Inclusion and exclusion criteria applied to the TECH (target user, evaluation focus, connectedness, and health domain) framework.

Domain	Inclusion criteria	Exclusion criteria
Target user	Parents or caregivers of newborns up to infancy (aged 0-12 months) Individuals or families	Health care professionals, nursery or care providers, or organizations
Evaluation focus	Quality, functionality, accessibility, and adherence to best practice guidelines	Privacy (do not expect personal data to be collected)
Connectedness	Stand-alone	Connected to wearable devices, EHRs^a^, and health care services
Health domain	Safer sleep apps (eg, sleep positions, bed set-up, sleep environment, and cosleeping) Educational or informational	Sleep trackers, feeding, milestones, or sound machines

^a^EHR: electronic health record.

A preliminary search was conducted using no limits or filters and the following key terms: safe sleep, safe sleep guidance for babies, and safe sleep guidance for infants, on the UK Apple App Store and Google Play Store. This confirmed that the number of apps available was manageable for the review and ensured that relevant apps were not missed. These same search terms were used for the final search conducted between January 6 and 13, 2026. The search was done manually, without the use of automated tools.

The name and developer of each app were manually recorded in a Google Sheets spreadsheet. Three lists of apps were produced following the 3 key term searches, and deduplication of apps was conducted separately by both researchers across the keyword searches. One author then screened each app’s eligibility based on its name and the written and image descriptions on the respective app store to assess whether it met the eligibility criteria. A second screening and deduplication stage then took place to identify duplicates between the stores and to screen eligibility after each app had been downloaded. Depending on platform availability, researchers downloaded each app onto 1 of 2 devices: an iPhone 11 (iOS system) and a Samsung S22 (Android system). Two researchers (NM and SY) then conducted independent eligibility assessments and reached a consensus. A third researcher (NG) helped resolve any disagreements. The screening of downloaded mHealth apps was conducted by both researchers on the same day to avoid changes in app store search results, which can vary from day to day. A flow diagram was developed to depict the search and screening process.

Apps available on both iOS and Android platforms were retained only once, to determine their multiplatform availability and to confirm which version was the most recent. Only 1 multiplatform app was included in the review, and this was evaluated on the Android device.

### Data Extraction

Data were extracted into a predefined data extraction (coding) sheet created within Google Sheets. Descriptive characteristics were extracted, including app name, developer, version number, the app stores where they were available, cost of in-app purchases, size, average user rating, number of user ratings, and affiliation (name of professional health, medical, clinical, or government body affiliated with the app).

Quality was assessed using the 19-item Mobile App Rating Scale (MARS), which evaluates 4 dimensions: engagement, functionality, aesthetics, and information quality [24]. Each item was rated using a 5-point Likert scale: inadequate (1 point), poor (2 points), acceptable (3 points), good (4 points), and excellent (5 points). The authors evaluating the apps (SY and NM) used the step-by-step training video on YouTube to aid their use of MARS and received additional training from the supervising author (NG). The subjective quality scale was excluded to ensure that all evaluations remained fully objective.

Functionality was assessed using the IMS Institute for Healthcare Informatics Functionality Scale, with each of the following functions rated 1 (present) or 0 (absent): inform; instruct; record, collect data, share data, evaluate, intervene; display; guide; remind or alert; and communicate [25]. The researchers also recorded the presence of AI as present (n=1) or absent (n=0). A written summary was made of any AI features.

Readability metrics were used to evaluate accessibility features, including the Flesch-Kincaid metrics integrated within Microsoft Word. The Flesch Reading Ease score provided a value between 0 and 100, where higher scores reflect that the content is easier to read [26]. Alongside this, the Flesch-Kincaid Grade Level offered an approximate US school grade required to understand the text [27]. It was recorded whether the app enabled users to change the language and whether dark or night mode features were available, either by reflecting the default phone settings or allowing in-app adjustments. These features were considered important for parents using the app during nighttime care, as they may help to minimize sleep disruption.

Each app’s adherence to best practice guidelines for safer sleep and cosleeping, defined as any shared sleep between an adult and a baby (eg, on a bed, sofa, or chair) [8], was evaluated using bespoke criteria informed by evidence-based guidance [4,5,28]. The presence of the following information was rated as present (n=1) or absent (n=0) and then summed for a total score:

- Lie your baby on their back
- Keep their cot clear
- Use a firm, flat, and waterproof mattress
- Recommend against bed-sharing (shared sleep between an adult and a baby on an adult bed for the majority of the night) if the baby has a low birth weight, or either parent has had 2 or more units of alcohol, smokes, is taking medication that causes drowsiness, or has used recreational drugs
- Avoid your baby getting too hot
- Sleep your baby in the same room as you for at least the first 6 months
- Do not sleep on a sofa or chair with the baby
- If bed-sharing: do not have pillows or duvets near the baby
- If bed-sharing: do not have other children or pets in the bed

### Data Synthesis and Analysis

Descriptive statistics were calculated in Microsoft Excel for all relevant items related to app characteristics, quality, functionality, accessibility, and adherence to best-practice guidelines. Text-based summaries (eg, description of any AI features) were summarized using a content synthesis approach. Results were presented using tables and figures (eg, a radar graph), where applicable. The apps were cross-compared using criteria related to quality, functionality, and adherence to best-practice guidelines to identify the highest-scoring apps.

Interrater reliability was also calculated to determine levels of agreement between the researchers conducting the quality and functionality evaluations using MARS and the IMS Institute for Healthcare Informatics functionality scores. These were calculated using IBM SPSS. We calculated an intraclass correlation coefficient for all MARS items using an absolute-agreement 2-way mixed-effects, average-measures model [29]. Interpretations were as follows: <0.5 (poor), 0.5-0.75 (moderate), 0.75-0.90 (good), and >0.90 (excellent) [30]. As the IMS Institute for Healthcare Informatics functionality score uses a binary measure (categories: present=1 and absent=0), we calculated Cohen κ. Interpretations of agreement were <0 (poor), 0.01-0.20 (slight), 0.21-0.40 (fair), 0.41-0.60 (moderate), 0.61-0.80 (substantial), and 0.81-1.00 (almost perfect) [31].

## Results

### App Market Search and Screening

**Figure 1:**
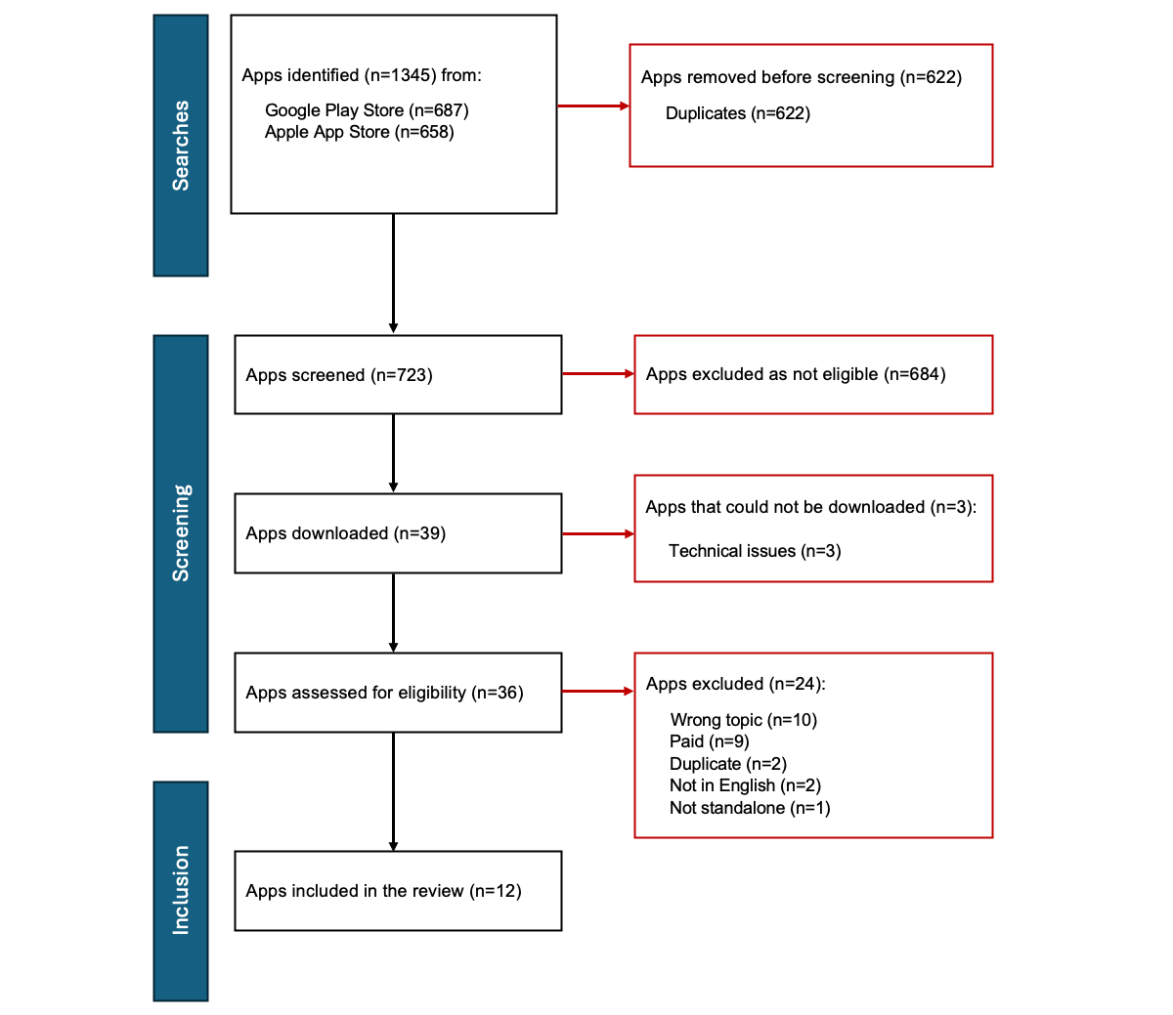
CAPPRRI (Consensus for APP Review Reporting Items) flowchart presenting the app search and screening process.

The initial search yielded 1345 apps. A total of 622 duplicates were removed: 297 from the Google Play Store, 286 from the Apple App Store, and an additional 39 identified between the 2 stores. A total of 723 apps were screened based on their names and the text and graphic descriptions in the app stores, leading to the exclusion of 684 apps as they did not meet the eligibility criteria. After this, 39 apps were downloaded for the second screening stage; however, 3 did not open or could not be accessed. Apps were excluded for the following reasons: addressing the wrong topic (n=10), requiring paid subscriptions or payments to access in-app content and features (n=9), not being in English (n=2), and not being stand-alone (n=1). Two others were duplicates; however, because the apps and developers had different names, they were not identified as duplicates during the initial screening stage. As a result, 12 apps were included in the review. Figure 1 presents the CAPPRRI flowchart summarizing the app search and screening process for the review.

### Descriptive Characteristics

Of the 12 apps included in the review, 50% (n=6) were available on the Apple App Store, and 42% (n=5) were available on the Google Play Store; only 1 (8%) was available on both. All were free to download, although 5 (42%) contained in-app purchases to unlock premium or additional content. Two (17%) apps were affiliated with a professional health, medical, clinical, or government body. The average app size was 99.34 (SD 54.10) MB, ranging from 17.70 MB to 232.70 MB. Of the 10 apps that had been rated by users, the total number of user ratings was 571,016, ranging from 1 to 340,000. An average user rating score of 4.11 out of 5 was calculated. Table 2 provides a summary of the app characteristics, and Multimedia Appendix 2 provides a list of all 12 apps and a plain language summary of the review.

**Table 2:** Key characteristics and content of the reviewed apps (N=12).

Characteristic		Value
**Market availability, n (%)**		
	Apple Store	6 (50)
	Google Play Store	5 (42)
	Both	1 (8)
**Cost, n (%)**		
	Free to download	12 (100)
	In-app purchases	5 (42)
**Affiliated with a professional health, medical, clinical, or government body, n (%)**		
	Yes	2 (17)
	No	10 (83)
**The user can change the language, n (%)**		
	Yes	0 (0)
	No	12 (100)
**The user can change to night/dark mode, n (%)**		
	Yes	3 (25)
	No	9 (75)
**Sleep guidance mentioned, n (%)**		
	Lie your baby on their back	12 (100)
	Keep their cot clear	10 (83)
	Use a firm, flat, and waterproof mattress	11 (92)
	Recommend against bed-sharing (shared sleep between an adult and a baby on an adult bed for the majority of the night) if the baby has a low birth weight, or either parent has had 2 or more units of alcohol, smokes, is taking medication that causes drowsiness, or has used recreational drugs	11 (92)
	Avoid your baby getting too hot	12 (100)
	Sleep your baby in the same room as you for at least the first 6 months	10 (83)
	Do not sleep on a sofa or chair with the baby	8 (67)
	If bed-sharing: do not have pillows or duvets near the baby	4 (33)
	If bed-sharing: do not have other children or pets in the bed	3 (25)
**Readability**		
	Reading ease score, mean (SD)	76.6 (SD 7.98)
	Reading age (US grade level)	6.9

### Sleep Guidance

While all 12 apps contained some information regarding safer sleep practices, this was addressed and named differently (eg, calling it cot death, SIDS, or safe sleep). Of the 9 criteria pertaining to safer sleep best practice guidance, the apps mentioned an average of 6.75 criteria (SD 1.71; range 3-9). Only 2 apps mentioned all the criteria.

All 12 apps mentioned placing the infant on their back as well as avoiding the baby getting too hot. Additionally, 92% (n=11) of the apps addressed the importance of using a firm, flat, and waterproof mattress and 92% (n=11) recommended against bed-sharing (shared sleep between an adult and a baby on an adult bed for the majority of the night [8]) if the baby has a low birth weight or either parent has had 2 or more units of alcohol, smokes, is taking medication that causes drowsiness, or has used recreational drugs. Most (10/12, 83%) mentioned keeping the cot clear and sleeping in the same room as the baby for at least the first 6 months. However, only 33% (n=4) mentioned not having pillows or duvets in the bed if parents choose to bed-share, and only 25% (n=3) highlighted the importance of not having any other person or pet in the bed if parents choose to bed-share. Table 2 presents the criteria and how many apps mentioned each one.

Two (17%) of the apps contained information that contradicts safer sleep guidance. Both highlighted practices such as swaddling and cosleeping with no additional information on how to do so safely, for example, not swaddling above the shoulders, keeping swaddles loose around the hips, and only cosleeping with 1 adult in the bed and no pillows or blankets.

### Accessibility

The average reading ease score was 76.6 (SD 7.98; range 62.90-91.40). All apps scored above 60, meaning that most adults should be able to understand the content. The average reading age was US grade level 6.9, ranging from the fifth grade to the ninth grade. However, 1 app exclusively provided content in video format, and therefore, readability scores could not be generated. None of the apps provided the ability to change the language (beyond English), and only 3 (25%) could be switched to night/dark mode either through app settings or by automatically adapting to the device’s settings.

### Functionality

The IMS Institute for Healthcare Informatics functionality score was used to identify functions within each app. There was moderate agreement between the 2 raters (κ=0.494, 95% CI 0.353-0.635; *P*<.001). Consensus was met on all items.

**Figure 2:**
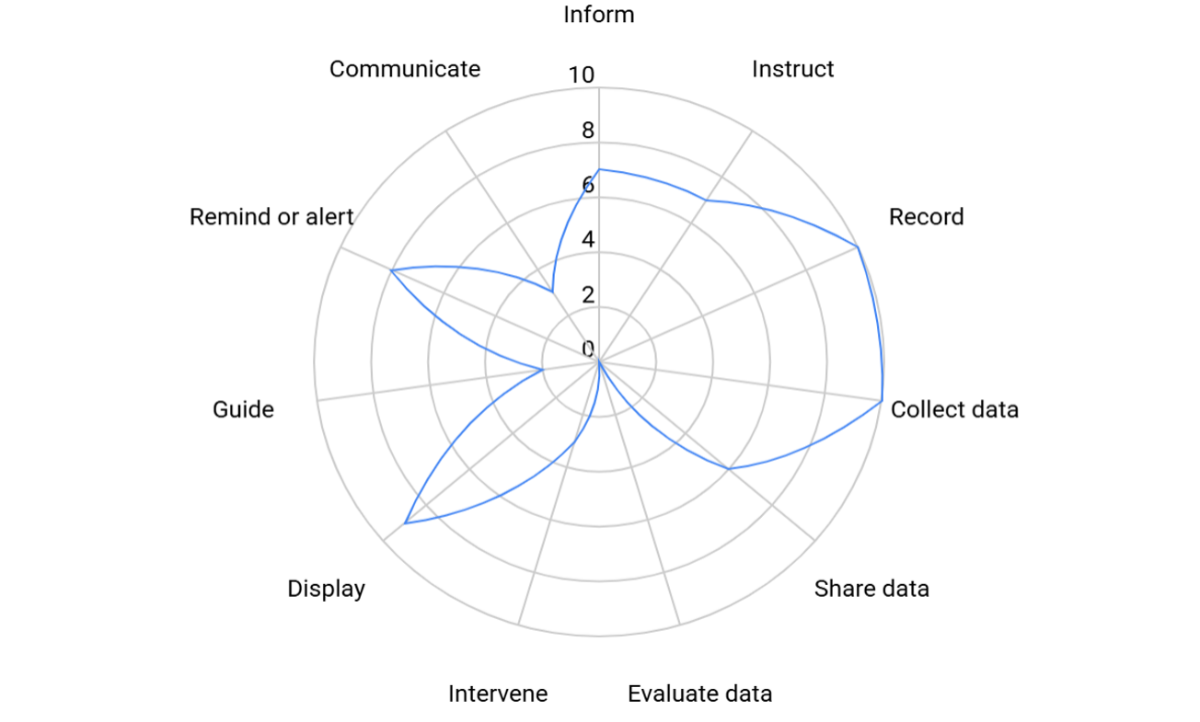
Radar graph showing app functionality using the IMS Institute for Healthcare Informatics functionality score for 11 items.

The apps had an average of 5.4 (SD 2.5) functions. The range of functions was from 2 to 9. The 3 most common functions were record (10/12, 83%), collect data (10/12, 83%), and display (9/12, 75%). The 4 least common functions were evaluate data (0/12, 0%), guide (2/12, 17%), and communicate and intervene (both 3/12, 25%; Figure 2).

A total of 2 apps (Baby Buddy: Pregnancy & Parent and Pregnancy Tracker & Baby App) had 9 out of the 11 functions, and 3 apps (Little One, Baby+ Your Baby Tracker, and Infant sleep info) had 7 functions. There were 2 apps (SIDS Info and Baby Sleep Magic) that only had 2 out of the 11 functions. The remaining apps ranged from having 3 to 6 functions.

Only 1 (8%) app contained an AI assistant. The application, “Bounty: Pregnancy and Baby App,” featured a “friendly AI Midwife Assistant” that was available to answer questions until a human midwife could answer between Monday and Friday from 10 AM to 2 PM.

### Quality

All apps underwent an independent evaluation carried out by 2 raters using MARS. There was excellent agreement between the raters (intraclass correlation coefficient 0.96, 95% CI 0.948-0.969; *P*<.001).

The mean overall quality score was 3.7 (SD 0.43; range 2.92-4.13). Almost all applications, 92% (11/12) met the minimum acceptability score of 3. The apps “Little Ones” and “Baby Buddy: Pregnancy and Parent” had the highest overall rating (4.1), and “Asianparent: Pregnancy & Baby” had the worst rating (2.9). Mean scores for engagement, functionality, aesthetics, and information quality were 3.3 (SD 0.56; range 2.4-4.3), 4.1 (SD 0.58; range 2.88-5.00), 3.6 (SD 0.73; range 2.67-4.67), and 3.8 (SD 0.68; range 2.38-4.80), respectively. Half of the apps (6/12, 50%) scored the highest on the functionality dimension, 4 apps (33%) scored the highest on the aesthetics dimension, 2 (17%) scored the highest on the information quality dimension, and none scored the highest on the engagement dimension. Three apps (25%) scored the lowest on the engagement dimension, 1 (8%) scored the lowest on the functionality dimension, 4 (33%) scored the lowest on the aesthetics dimension, and 3 scored the lowest on the information quality dimension.

Only 2 (17%) apps, “Baby Buddy: Pregnancy & Parent” and “Bounty: Pregnancy and Baby App,” had been formally trialed or tested. However, one yielded positive or partially positive results [32,33] while the other received critical results for its content as it provided contradictory information around avoiding alcohol consumption but also limiting this to 1-2 units once or twice per week [34]. It is important to note that we did not verify whether this contradiction has been amended or whether the app has been updated. None of the apps had been tested in randomized controlled trials.

### Cross-Comparison of Highest-Scoring Apps

A cross-comparison of the apps with the highest IMS functionality and MARS scores was undertaken. The highest performing apps and their safer sleep guidance score (out of 9) are presented in Table 3.

**Table 3:** The highest performing apps included in the review.

App; developer (version)	Cost; platform	IMS score^a^	Total MARS^b^ score	Purpose	Safer sleep guidance criteria^c^
Pregnancy Tracker & Baby App; What to Expect (version 7.81)	Free; Google Play Store	9	4.13	Baby growth tracker that provides general advice and guidance around pregnancy and infancy	7
Baby Buddy: Pregnancy & Parent; Babyzone (version 1.2.0)	Free; Google Play Store	9	4.09	Provides personalized information and guidance through pregnancy and infancy	8
Little Ones; Little Ones (version 7.6.4)	GBP £59- GBP £99^d^ (in-app); Apple App Store	7	4.09	Provides a comprehensive sleep program for babies and infants	6

^a^IMS Institute for Healthcare Informatics functionality score (maximum score=11).

^b^MARS: Mobile App Rating Scale; maximum score=5.

^c^Safer sleep guidance criteria score (maximum score=9).

^d^GBP £1=USD $1.32 as of June 26, 2026.

## Discussion

### Overview

This systematic mHealth app review found that there were 12 stand-alone safer sleep apps free to download for parents and caregivers of newborns up to infancy in the United Kingdom. Almost all met the minimum acceptability criteria for quality. The apps ranged in functionality, with an average of 5.4 functions each, although only 1 had an AI feature. A total of 11 apps had written content and could be considered to contain accessible language, as demonstrated by reading ease scores of more than 60. However, none of the apps provided the option to change the language, creating a barrier for people who cannot speak or read English, and very few could change to night/dark mode, limiting usability in dark rooms. Regarding alignment to best practice guidelines, the apps mostly did not provide comprehensive content—most met at least half of the criteria, but only 2 presented all the criteria. Some apps also promoted unsafe practices.

This review showed that there is a limited number of safer sleep guidance apps available across both stores. It was challenging to find apps that contained safer sleep guidance because of the vast number of apps catering to babies, infants, and sleep, many of which were simply sleep trackers, white noise apps, alarms, or baby growth trackers. A large percentage of the apps reviewed were not limited to safer sleep guidance apps, with only 1 focusing solely on providing safer sleep guidance. The others also included general baby and infant safety advice, and many were growth and sleep trackers with guidance sections focusing on safer sleep as a small component. This finding is consistent with a previous review of the app market in Germany, finding that the content of most sleep apps was lullabies, music, or songs, and sleep information was very rare [35]. Similarly, Ejezie et al [36] concluded that digital health strategies for SIDS prevention remain limited, and mostly include online interventions (eg, web-based modules).

Although almost all of the apps we reviewed (11/12, 92%) did meet the minimum acceptability criterion for quality, only 2 of them managed to fully adhere to best practice guidelines as outlined by established UK health bodies, including NICE [28], the NHS [4], and the Lullaby Trust [5]. A few also mentioned the use of techniques that did not align with best practice, such as swaddling, and some did not include further guidance on how to do so safely. The aforementioned review conducted by Schlarb et al [35] also found that there was only a small percentage of apps available in Germany related to sleep information for parental behavior. An analysis of web search results by Chung et al [37] related to safer sleep recommendations found that only 43.5% of the 1300 websites provided accurate information. This further emphasizes the gap in accurate, safer sleep guidance and recommendations observed through this review.

Furthermore, it is important to note that although only 1 of the apps reviewed in this study used AI, this feature is increasingly being incorporated into mHealth apps [38], which raises additional considerations around data privacy and the uncertainty of AI-generated results (especially when delivering personalized medical care) [39].

Not many of the apps had been trialed or tested, and so it remains unclear whether the reviewed apps are effective in promoting safer sleep behaviors and in reducing SIDS. Previous research has shown that although families are often aware of safer sleep guidance, this does not always translate into practice, especially when the guidance is impractical to implement [40-42]. Information-only resources (including those delivered via smartphone apps) may therefore have limited effectiveness if not provided with contextual support. There has been 1 randomized controlled trial conducted by Moon et al [43] with 1263 mothers in the United States, finding that the intervention group receiving the mHealth intervention with regular text and email messages and videos had significantly higher rates of safer sleep practice compared to the control group. However, the authors point to further research needed to assess long-term impact in order to establish whether adherence to best practice sleep guidance contributes to a reduction in SIDS and whether widespread implementation is possible [43]. The requirement for further research on whether engagement with mHealth interventions correlates with changes in safer sleep practices is also emphasized by Krishnamurti et al [44], who integrated safer sleep information into an existing app, and Aggelou et al [45], who systematically reviewed 23 studies on educational safer sleep interventions targeting caregivers.

It is also important to explore the impact of these interventions in the context of parents receiving alternative advice, as well as on their effects on parental attitudes and norms regarding safer sleep recommendations. This review recognizes that adherence to safer sleep guidance can become secondary when dealing with the responsibilities of caring for a newborn, and parents who opt to bed-share do so for a multitude of reasons, among them to provide comfort, for bonding, and for better/more sleep for both the infant and parent [46]. Additionally, previous research from Australia, New Zealand, and internationally has highlighted that cosleeping (including bed-sharing) is common [42,47,48], either intentionally or unintentionally, during feeding or periods of exhaustion [47,49]. As a result, many families develop their own safer sleep strategies, such as choosing to sleep on a chair rather than in a bed [40,49]. Our research reflects this broader issue, as three-quarters of the apps did not provide strategies for safer bed-sharing, despite the likelihood that many families will encounter these situations. Current apps available in the United Kingdom, therefore, do not adequately support parents across the full spectrum of real-world caregiving scenarios, including unintentional shared sleep and culturally normalized bed-sharing contexts.

Consequently, it is essential to provide context-specific support to families and assess alternative advice given to parents who cannot or do not follow safer sleep guidance, for example, how to safely cosleep or swaddle [43]. Ball and Keegan [50] also highlight that parents often use social media to share information around sleep and this, along with infant sleep apps, may influence cultural narratives around nighttime care. They caution that costly digital sleep technologies may undermine responsive parenting practices and capitalize on parental anxiety. Evidence also suggests that mHealth interventions can enhance adherence to safer sleep guidance by positively influencing maternal attitudes and social norms related to specific recommendations (eg, supine sleeping and room-sharing without cosleeping) [51]. Research is warranted in these areas.

### Implications

As outlined above, research is needed to determine the long-term impacts of mHealth apps on preventing SIDS and to explore the context in which parents receive and follow safer sleep guidance. However, there are also several implications for app developers and established safer sleep organizations.

As already discussed, many of the apps were not limited to providing only safer sleep advice. It is therefore important to acknowledge that many people will not be interested in downloading an app that simply contains information on safer infant sleep, as this can already be found online. Rather than developing new apps, we therefore recommend that established organizations such as The Lullaby Trust [5,8] incorporate safer sleep information into the apps they have developed and manage. This is because the organization is a recognizable and trusted name in the United Kingdom that many health care workers already signpost new parents to. For example, their app “Lullaby Trust Baby Check” is already used by parents to determine whether their baby needs to see a health care professional. In a review of apps for managing acute childhood illness, this app was among the highest rated for aligning with end user requirements and was found to already support parents in the United Kingdom [52]. Another service evaluation found that most health visitors were already familiar with it and recommended it to parents of infants up to 6 months old [53]. Interestingly, and consistent with our findings, the evaluation found that health visitors wanted the app to be translated into other languages [53]. It is vital that any content for diverse communities be co-designed with them, given that there are deep-rooted cultural and traditional sleep practices that must be considered. A brief section on safer sleep (including how best to support families who are already bed-sharing) within the app could also leverage The Lullaby Trust’s reputation and help reduce digital waste by reducing the need for a new app to be developed.

Although non–app-based tools such as dedicated websites exist, it can be more convenient to have a smartphone app that already contains evidence-based information regarding safer sleep, particularly as this removes the burden of finding information and may be used offline. Developers who are considering creating apps about infancy or improving their existing apps should include engaging features that provide advice and guidance around safer sleep practices. For example, an American project leveraged simple push notifications in order to increase engagement with safer sleep and breastfeeding education modules within a pregnancy app, which was found to be particularly effective in engaging parents living in high-risk zip codes where SIDS education is most likely to have an impact [44]. When producing this content, developers should partner with health and care workers who have expertise in safer sleep practices and can translate the best practice guidance into easy-to-understand (and reliable) content. Information could be presented in a variety of ways to accommodate different visual and auditory preferences, for example, diagrams, pictures, videos, audio recordings, and writing. This, along with the ability to change languages and to switch to nighttime mode, is important to ensure maximum accessibility and engagement.

It is important to acknowledge that the criteria used to evaluate safer sleep apps in this review take a primarily risk elimination approach, strongly advising against cosleeping. A review by Kruse et al [54] on safer sleep messaging in Australia also highlighted that less than half of the organizations they reviewed provided strategies to engage in safer shared sleep and acknowledged that shared sleep may occur unintentionally or be considered a familial, cultural, or logistical preference. Families often do not disclose cosleeping to health care practitioners [49], potentially due to anticipating judgment or disapproval. A shift toward sensitive and consistent messaging that better reflects the realities of baby sleeping is needed, and this should ideally employ a risk minimization strategy, acknowledging that cosleeping does occur and that risks can be minimized [54,55]. This approach should be used in the future to evaluate safer sleep apps and the content within these apps.

### Strengths and Limitations

This review followed a structured and transparent 7-step process informed by appropriate frameworks, while validated measures were used to evaluate quality, functionality, and readability. Two raters were involved in the screening and evaluations, with strong agreement. The review was further strengthened by examining adherence to evidence-based UK safer sleep guidance.

Limitations included the restriction to free-to-download apps available on the UK Apple App Store and Google Play Store at the time of the search, which may not capture apps available in other regions, other stores, or that have since become available. Additionally, conclusions regarding the impact of these apps on safer sleep practices cannot be drawn, as the review did not assess changes in parental behavior or downstream outcomes. It is therefore unclear whether the highest-scoring apps are the most effective.

### Conclusion

This systematic mHealth app review identified a limited number of safer sleep guidance apps for parents and caregivers of newborns and infants across the 2 app stores in the United Kingdom. Most of these met the minimum acceptability criteria for quality and contained accessible written content. However, most of the reviewed apps did not provide comprehensive safer sleep guidance, and it was concerning to note that some encouraged practices that are against best practice guidelines. Further long-term research is needed on the effectiveness of mHealth interventions in improving adherence to safer sleep guidance and their ability to reduce SIDS. Information related to best practice guidance around safer sleep and preventing SIDS should be included in established newborn and infant apps, in partnership with trusted organizations, and should be available in several languages. This may result in the development of safer sleep guidance applications that are both trusted and accessible.

## Appendix

Multimedia Appendix 1 CAPPRRI checklist.

Multimedia Appendix 2 Additional table and the plain English summary.

## Abbreviations

AI: artificial intelligence
CAPPRRI: Consensus for APP Review Reporting Items
ICD-11: International Classification of Diseases, 11th Revision
MARS: Mobile App Rating Scale
mHealth: mobile health
NHS: National Health Service
NICE: National Institute for Health and Care Excellence
SIDS: sudden infant death syndrome
TECH: target user, evaluation focus, connectedness, and health domain
