# Supplementary material for: Safer Sleep Guidance in Standalone UK Smartphone Apps for Parents of Newborns and Infants: Systematic mHealth App Review
Source: JMIR Mhealth Uhealth. 2026 Jul 10;14:e95642. doi: 10.2196/95642 (PMC13401075; doi:10.2196/95642)
Supplement: Multimedia Appendix 1 [file mhealth_v14i1e95642_app1.pdf]

CAPPRRI
CONSENSUS FOR APP REVIEW REPORTING ITEMS

Consensus for APP Review Reporting Items (CAPPRRI) guidance

Note: The CAPPRRI guidance was under development when this manuscript was reported. Some of the below items are therefore subject to change.

SECTION	ITEM	CAPPRRI GUIDANCE ITEM	LOCATION REPORTED*
**TITLE**			
Title	1	Identify the review as a systematic app review.	Title
**ABSTRACT****			
Abstract	2	State the review's review/research question, and/or aim.	Abstract
	3	State the eligibility criteria for the included apps.	Abstract
	4	Specify the app stores (and if relevant, databases) and search terms used to identify and retrieve apps, the stores' geographical location (country), and date/s of searching.	Abstract
	5	Name the method/s used to evaluate the apps, extract and analyse/synthesise the data for the app evaluation (e.g., descriptive statistics, content synthesis).	Abstract
	6	Specify the total number of apps reviewed and summarise relevant characteristics (e.g., platforms where available, care focus, language and costs of use).	Abstract
	7	Present the results for the main outcomes of the app review in line with the review's aim and objectives (e.g., usability evaluation, mean quality scores, adherence to clinical guidelines or mean number of behaviour change techniques present).	Abstract
	8	Interpret the results and outline key implications. If naming or recommending specific apps, include the developer's name and the app's version number.	Abstract
**PLAIN LANGUAGE SUMMARY*****			
Plain language summary	9	Present a summary of the review written in plain language. Only if relevant and appropriate, name up to five of the best rated apps, detailing the criteria used to make this judgement and key information (e.g., target/intended users, cost, country and platform available, purpose, developer and version number). Specify the date or period when the evaluation was undertaken and a summary of who was involved in the review.	Plain language summary
**INTRODUCTION**			
Introduction	10	Describe the rationale for the review in the context of existing knowledge.	Background
**AIM/ RESEARCH QUESTION / OBJECTIVES**			
Aim, Research Question, Objectives	11	State the reviews' aim or review/research question, and objective/s. Also state how this was framed (e.g., guided by a framework or existing evidence).	Abstract, Background , Methods

SECTION	ITEM	CAPPRRI GUIDANCE ITEM	LOCATION REPORTED*
**METHODS**			
Protocol and registration	12	Report whether a protocol was developed, where it is available (name of register and registration ID, URL, or citation). Outline any amendments made from the protocol before review or deviations during review. If the app review was not registered or archived, state this.	Methods
Researcher/ team experience and stakeholder engagement	13	Outline who conducted the review and any relevant experience which may enhance its credibility.	Methods
		If applicable, explain any additional stakeholder engagement and consultation, e.g., who was included, in what capacity, what training they received and what insight they brought to the review.	N/R
Eligibility criteria	14	Specify the criteria for assessing the relevance of apps for inclusion (or exclusion) in the review.	Methods
Searches	15	Present the full search strategies for all sources used to identify and retrieve apps, including the app stores/platforms (and if relevant, academic databases) searched, the date of searches, the geographical location (country) of the app markets/platforms, any keywords, filters and limits used. State whether searches were conducted manually or using an automated tool. At minimum, the main text should report a summary of the search strategy, with further details provided in a supplementary file.	Methods
Screening	16a	Specify the procedure for deciding whether an app met the eligibility criteria for the review, including how many reviewers screened each app, and whether they worked independently.	Methods
	16b	Provide details of any tools/software used, including any artificial intelligence (AI) enabled tools.	Methods
	16c	Explain the duplicate removal process including,<br>i) How duplicates were viewed/identified (e.g., app store pages, screen grabs, Excel).<br>ii) What information was used to determine if apps were duplicates (e.g. developer, version numbers).	Methods
	16d	For multiplatform apps (i.e., apps that are available in multiple platforms), state which app was included and provide a rationale.	Methods
Evaluating the apps: devices	17	State the device model and correlated version of the operating system used when evaluating the apps.	Methods
Evaluating the apps: assessment metrics	18	Describe (and cite) the data extraction items and assessment metrics selected in relation to the research questions of the review, and the frameworks and/or instruments/evaluation measures applied for their extraction.<br>i) If relevant, outline, cite and justify modification to or use of validated evaluation measures.<br>ii) If relevant, outline, cite and justify use of bespoke measures.<br>iii) If considering efficacy, effectiveness, equity or implementation outcomes, state how these were addressed and whether evidence	Methods: Data extraction

SECTION	ITEM	CAPPRRI GUIDANCE ITEM	LOCATION REPORTED*
		reported from academic institutions, end user feedback, or research studies (e.g., clinical research or randomised controlled trials) was drawn on.<br>iv) If considering adherence to clinical guidelines, name, justify and cite these appropriately.	
Evaluating the apps: reviewers and methods used to extract data	19a	Specify the methods used to collect data about the apps (i.e., evaluate them), including how many reviewers collected data about each app, and whether they worked independently or in duplicate.	Data extraction
	19b	Specify the methods used to assess the reliability of the app evaluation (e.g., calculate inter-rater reliability of the app evaluation measures).	Data extraction
	19c	Provide details of any tools/software used.	Data extraction
Data analysis	20	Describe any methods used to synthesise and/or analyse the data and justify the use of that method	Data synthesis/ analysis
**RESULTS**			
Search and screening	21	Describe the results of the app search and screening process, stating how many apps were considered at each stage. The description should be presented as a flow diagram and with text.	Results: App market search and screening
Characteristics of reviewed apps	22	Name the reviewed apps (and their version number and developer) in-text or in a supplementary file and present their key characteristics (e.g., main purpose, cost, size (MB), platform available, target population, privacy and security measures and number of downloads).	Suppl file
Inter-rater reliability	23	If relevant, present results of inter-rater reliability calculations for the app evaluations (e.g., Cohen's Kappa, Intraclass Correlation Coefficient, Kendall's coefficient of concordance or raw agreement (%)).	Results
Syntheses/ analyses	24	Provide results of all syntheses/analyses conducted, including descriptive and inferential statistics (if relevant).	Results: Descriptive characteristics
Recommendations ****	25	If relevant to the aim of the review, name and/or describe the best rated apps, detailing the criteria used to make this judgement and their key characteristics. If relevant, state how adherence to clinical guidelines or evidence of specific benefit from academic institutions, end user feedback, or research studies has informed the recommendation.	Results: Cross-comparison of highest scoring apps
**DISCUSSION**			
Summary and discussion of results	26	Summarise and interpret the results in the context of existing evidence.	Discussion

SECTION	ITEM	CAPPRRI GUIDANCE ITEM	LOCATION REPORTED*
Strengths and limitations	27	Discuss limitations and strengths related to the review methods (e.g., search/screening of app databases, evaluation of the apps or analysis).	Discussion
Implications	28	Provide a 'so- what' analysis, discussing implications of the results for practice (including healthcare, technology development, use or implementation), policy, and future research (including app review methods).	Discussion
**OTHER**			
Support and funding	29	Acknowledge sources of support (financial or otherwise) for the conduct and publishing of the review.	Declaration s
Conflicts of interest	30	Declare any relevant conflicts of interest.	N/R-declarations
Review resources	31	If relevant, explain where app review resources can be accessed e.g., data collection forms, data from the app evaluation measures or any other material.	In-text; refer to suppl file
