# Supplementary material for: Safer Sleep Guidance in Standalone UK Smartphone Apps for Parents of Newborns and Infants: Systematic mHealth App Review
Source: JMIR Mhealth Uhealth. 2026 Jul 10;14:e95642. doi: 10.2196/95642 (PMC13401075; doi:10.2196/95642)
Supplement: Multimedia Appendix 2 [file mhealth_v14i1e95642_app2.docx]

**Supplementary file**

Table 1. All the apps included in the review (N=12).

| **Name** | **Developer** | **Version number** | **Platform available** | **IMS Score (of 11)** | **Total MARS score (of 5)** | **Sleep guidance score (of 9)** |
| --- | --- | --- | --- | --- | --- | --- |
| Baby Sleep Coach | Appulize | 1.1.0 | Apple App Store | 3 | 3.02 | 3 |
| Baby Sleep Magic | Baby Sleep Magic Pty Ltd | 2 | Apple App Store | 2 | 3.32 | 6 |
| Bounty: Pregnancy and Baby App | Bounty Joy Limited | 2.27.33 | Apple App Store | 6 | 3.75 | 9 |
| Little Ones TM | Little OnesTM | 7.6.4 | Apple App Store | 7 | 4.09 | 6 |
| Lovebug | Lovebug | 7.8.0 | Apple App Store | 6 | 3.55 | 8 |
| SIDS info | Zyndo LLC | 4.2 | Apple App Store | 2 | 4.03 | 9 |
| Baby Buddy: Pregnancy & Parent | Babyzone | 1.2.0 | Google Play | 9 | 4.09 | 8 |
| Baby+ Your Baby Tracker | Philips Electronics UK Limited | 7.3 | Google Play | 7 | 4.06 | 7 |
| Newborn & You: Baby Tracker | We test it! by Radu Ola | 1.0.4 | Google Play | 3 | 3.42 | 6 |
| Pregnancy Tracker & Baby App | What to Expect | 7.81 | Google Play | 9 | 4.13 | 7 |
| Asianparent: Pregnancy & Baby | theAsianparent- largest pregnancy & parenting app | 3.1.12 | Google Play | 4 | 2.92 | 7 |
| Infant Sleep Info | Durham University | 11.34.0 | Google Play; Apple App Store | 7 | 3.57 | 5 |

**Plain language summary**

***What was this study about?***

This study looked at smartphone apps that give parents advice about how babies should sleep safely. We wanted to see what information the apps give, how good they are, and whether they follow trusted safer sleep advice. We also wanted to suggest how these apps could be improved.

***How was the study done?***

In January 2026, we searched the UK Google Play and Apple App stores for apps about safer sleep for babies under 12 months old. We looked at what each app included and how it worked.

Two students studying nursing and midwifery found and reviewed the apps. Both had training in safer sleep advice. They were supported by two experienced researchers in digital health, one of whom is also a registered nurse. The apps were scored for quality, functionality, accessibility and how closely they followed safer sleep guidelines.

***What did we find?***

We found 1,345 apps, but only 12 were actually about safe baby sleep. All were free to download, but some asked users to pay for extra features. Only a few apps were linked to health professionals or official organisations.

All 12 apps gave some safer sleep advice, but many did not include all the important points. Some apps gave advice that could be unsafe. Most apps were good enough in quality. The apps were all easy to read, but none could be used in languages other than English, and very few had a dark mode, which would be helpful when used at night.

***Which apps were the best?***

The three highest-rated apps were chosen based on how good they were overall and how many useful features they had. These apps gave better-quality information than most others, but they still did not include all safer sleep advice.

The top three apps were:

1. Pregnancy Tracker & Baby App (What to Expect) (Developer: What to Expect; Version: 7.81)
2. Baby Buddy: Pregnancy & Parent (Developer: Babyzone; Version: 1.2.0).
3. Little Ones™ (Developer: Little Ones™; Version: 7.6.4).

***What does this mean?***

There are apps that share information on safer baby sleep, but many do not give full or clear advice. Some even include unsafe tips. The apps could be improved by following all trusted safer sleep advice and being tested to make sure they really help parents keep babies safe.
